# Prospective Assessment of Cardiac Iron Deposition, Morphology, and Function by Magnetic Resonance Imaging in Non-Transfusion-Dependent and Neo-Transfusion-Dependent Thalassemia

**DOI:** 10.3390/jcm14124020

**Published:** 2025-06-06

**Authors:** Antonella Meloni, Laura Pistoia, Filomena Longo, Anna Spasiano, Valerio Cecinati, Elisabetta Corigliano, Zelia Borsellino, Ilaria Fotzi, Vincenzo Positano, Michela Zerbini, Stefania Renne, Luigi Barbuto, Alberto Clemente, Paolo Ricchi

**Affiliations:** 1 Bioengineering Unit, Fondazione G. Monasterio CNR-Regione Toscana, 56124 Pisa, Italy; positano@ftgm.it; 2 Unità Operativa Complessa Ricerca Clinica, Fondazione G. Monasterio CNR-Regione Toscana, 56124 Pisa, Italy; laura.pistoia@ftgm.it; 3 Dipartimento di Medicina Specialistica—Day Hospital della Talassemia e delle Emoglobinopatie, Azienda Ospedaliero-Universitaria Arcispedale “S. Anna”, 44124 Cona, FE, Italy; filomena.longo@ospfe.it; 4 Unità Operativa Semplice Dipartimentale Malattie Rare del Globulo Rosso, Azienda Ospedaliera di Rilievo Nazionale “A. Cardarelli”, 80131 Napoli, Italy; spasiano.anna@tiscali.it (A.S.); paolo.ricchi@aocardarelli.it (P.R.); 5 Struttura Semplice di Microcitemia, Ospedale “SS. Annunziata” ASL Taranto, 74123 Taranto, Italy; valerio.cecinati@als.taranto.it; 6 Ematologia Microcitemia, Ospedale San Giovanni di Dio—ASP Crotone, 88900 Crotone, Italy; krthal@libero.it; 7 Unità Operativa Complessa Ematologia con Talassemia, ARNAS Civico “Benfratelli-Di Cristina”, 90134 Palermo, Italy; zelia.borsellino@arnascivico.it; 8 SOC Oncologia, Ematologia e Trapianto di Cellule Staminali Emopoietiche, Meyer Children’s Hospital IRCCS, 50139 Firenze, Italy; ilaria.fotzi@meyer.it; 9 Diagnostica per Immagini e Radiologia Interventistica, Ospedale del Delta, 44023 Lagosanto, FE, Italy; m.zerbini@ausl.fe.it; 10 Struttura Complessa di Cardioradiologia-UTIC, Presidio Ospedaliero “Giovanni Paolo II”, 88046 Lamezia Terme, CZ, Italy; stefania.renne@virgilio.it; 11 Unità Operativa Complessa Radiologia Generale e di Pronto Soccorso, Azienda Ospedaliera di Rilievo Nazionale “A. Cardarelli”, 80131 Napoli, Italy; luigibarbuto@hotmail.com; 12 Department of Radiology, Fondazione G. Monasterio CNR-Regione Toscana, 56124 Pisa, Italy; clemente@ftgm.it

**Keywords:** cardiac size, iron overload, magnetic resonance imaging, neo-transfusion-dependent thalassemia, non-transfusion dependent thalassemia

## Abstract

**Objectives**: We compared changes in hepatic and cardiac iron levels, left ventricular (LV) and right ventricular (RV) dimensions and function, and bi-atrial areas, all assessed through magnetic resonance imaging (MRI), between patients with non-transfusion-dependent thalassemia (NTDT) and those with neo-transfusion-dependent thalassemia (neo-TDT) over an 18-month follow-up period. **Methods**: We included 32 NTDT patients (42.78 ± 12.62 years, 53.1% females) and 58 neo-TDT (>4 transfusions per year) patients (44.08 ± 14.13 years, 46.6% females), consecutively enrolled in the Extension-Myocardial Iron Overload in Thalassemia project. Iron overload was quantified by T2* technique, biventricular function and atrial areas by cine images. Macroscopic myocardial fibrosis was detected by the late gadolinium enhancement technique. **Results**: Changes in cardiac and hepatic iron levels, in biventricular ejection fractions, in LV mass index, and bi-atrial areas were comparable between the two groups. A trend of worsening biventricular dimensions was observed in the NTDT group, while the neo-TDT group showed an improvement (decrease) in biventricular size (LV stroke volume index: *p* = 0.036; LV cardiac index: *p* = 0.031; RV end-diastolic volume index: *p* = 0.034; RV stroke volume index: *p* = 0.033). The inter-group comparison showed significant differences in the changes of biventricular end-diastolic volume indexes (LV: *p* = 0.011 and RV: *p* = 0.034) and stroke volume indexes (LV: *p* = 0.036 and RV: *p* = 0.033) and in the cardiac index (*p* < 0.0001). At both MRI scans, the frequency of replacement myocardial fibrosis was comparable between the two groups. **Conclusions**: Our 18-month longitudinal data revealed distinct patterns of cardiac remodeling in NTDT and neo-TDT patients. The progressive ventricular dilation observed in NTDT patients highlights the need for careful MRI monitoring and potential interventions to address the long-term cardiac consequences of anemia.

## 1. Introduction

Thalassemia is a group of inherited hemoglobinopathies characterized by mutations in the genes responsible for producing the globin chains of hemoglobin, leading to ineffective erythropoiesis, hemolysis, and chronic anemia [1,2]. Non-transfusion-dependent thalassemia (NTDT) refers to the subset of thalassemia patients who do not require regular, lifelong blood transfusions [3,4]. This disease group encompasses a range of clinically diverse thalassemia forms, including beta-thalassemia intermedia (β-TI), hemoglobin E/β-thalassemia, and α-thalassemia intermedia [5]. NTDT patients typically experience mild anemia, and they may require occasional transfusion support during specific clinical situations, such as infection, pregnancy, or episodes of growth failure [4,5,6]. Over time, however, a significant proportion of NTDT patients may develop a need for regular blood transfusions as the disease progresses. Although not without risks [7,8,9,10], these transfusions are crucial in managing or preventing complications such as thrombosis, extramedullary hematopoiesis, pulmonary hypertension, leg ulcers, and cholelithiasis, and, in addition, they help reduce excessive gastrointestinal iron absorption [11,12,13,14,15,16,17]. The subset of NTDT patients requiring frequent transfusions is referred to as neo-transfusion-dependent thalassemia (neo-TDT) to distinguish them from individuals with long-standing transfusion-dependent thalassemia (TDT) [18].

Cardiac involvement is a significant component of the clinical picture of both NTDT and neo-TDT patients [17,19,20], though it has been relatively underexplored in the neo-TDT group. Cardiac magnetic resonance (CMR) is a powerful, non-invasive imaging technique that offers comprehensive assessments of both structural and functional cardiac abnormalities [21,22,23]. CMR’s high accuracy and reproducibility make it the gold standard for evaluating systolic function and cardiac chamber size [24]. Additionally, CMR can assess myocardial tissue composition, detecting changes such as edema or fibrosis [25,26], and it is uniquely capable of quantifying myocardial iron overload using the T2* technique [27,28].

Conversely to hepatic iron overload, myocardial iron overload has not been recognized as a significant clinical issue in NTDT patients [29,30,31,32,33,34]. Similarly, neo-TDT patients exhibit a low cardiac iron burden, likely due to the protective effect of residual expanded erythropoiesis, which may help prevent myocardial iron overload linked with transfusional iron accumulation [14,35,36].

However, recent CMR studies have shown that cardiac iron levels are higher in neo-TDT patients compared to NTDT patients [33,37]. On the other side, the same studies demonstrated a reduced ventricular dilation among neo-TDT patients, suggesting that blood transfusions may have a protective effect in mitigating the hemodynamic consequences of chronic anemia. The heart’s response to prolonged anemia involves compensatory mechanisms such as increased stroke volume and reduced peripheral vascular resistance, which result in ventricular dilation to accommodate the increased circulatory demands [38,39,40,41]. While these adaptations may initially maintain cardiac output, they place long-term stress on the heart, potentially leading to heart failure and other complications if not adequately managed.

The baseline comparisons of cardiac findings provide the foundation for assessing longitudinal trends in cardiac iron levels and function in a real-world clinical setting. This multicenter study aimed to compare the changes in cardiac iron deposition, size, and function between NTDT and neo-TDT patients over a follow-up of 18 months.

## 2. Materials and Methods

### 2.1. Study Population

The E-MIOT (Extension-Myocardial Iron Overload in Thalassemia) project is a nationwide network in Italy, consisting of 66 thalassemia centers and 15 MRI sites, where magnetic resonance imaging (MRI) image acquisition and analysis are carried out using a homogeneous, standardized, and validated approach [42]. All centers are connected through a web-based database that collects all clinical, instrumental, and laboratory data of the patients.

Eligibility for inclusion in the E-MIOT project was determined by the following criteria: (1) male and female subjects of any age diagnosed with thalassemia syndromes or structural hemoglobinopathies requiring MRI for the quantification of tissue iron overload; (2) provision of duly signed informed consent; (3) written consent authorizing the use and disclosure of protected health information; and (4) no absolute contraindications preventing the performance of the MRI examination.

We retrospectively included all 90 β-TI patients (43.62 ± 13.55 years, 44 females) consecutively enrolled in the E-MIOT project who performed an MRI follow-up study at 18 ± 3 months, according to the protocol. This schedule represents a compromise between the recommended monitoring intervals based on baseline cardiac iron levels [28] and the practical availability of MRI services in Italy.

Patients were categorized into two groups based on the transfusion regimen. The NTDT group was constituted by 32 (35.6%) patients. Of them, 13 patients were transfusion naïve, and 19 had received sporadic blood transfusions. The neo-TDT group included 58 (64.4%) patients who followed a regular transfusion regimen (>I4 transfusions per year), started at a mean age of 23.12 ± 19.83 years.

The study was conducted in accordance with the Declaration of Helsinki and received approval from the ethical committees of all MRI centers participating in the E-MIOT project. Informed consent was signed by all patients.

### 2.2. MRI

MRI exams were performed on clinical 1.5 T scanners (GE Healthcare, Milwaukee, WI, USA; Philips, Best, The Netherlands; Siemens Healthineers, Erlangen, Germany) equipped with phased-array coils. Breath-holding in end-expiration and ECG-gating were used.

The T2* technique was used for iron overload assessment. A mid-hepatic slice [43] and three parallel short-axis views (basal, medium, and apical) of the left ventricle (LV) [44] were obtained using gradient–echo multiecho sequences. Image analysis was performed by trained MRI operators (>10 years of experience) using a custom-written, previously validated software (HIPPOMIOT^®^, Version 2.0, Consiglio Nazionale delle Ricerche and Fondazione Toscana Gabriele Monasterio, Pisa, Italy, Year 2015). Liver T2* values were measured in a circular region of interest, traced in a homogeneous area of parenchyma, avoiding blood vessels [43], and were converted into liver iron concentration (LIC) values employing the most appropriate calibration curve (Wood’s calibration curve) [45]. The software provided the T2* value for the 16 segments of the LV, according to the American Heart Association (AHA)/American College of Cardiology (ACC) standardized myocardial segmentation [46]. An appropriate correction map was used to reduce distortions in segmental T2* values caused by magnetic susceptibility effects [47]. The global heart T2* value was calculated as the mean of the segmental measurements. A LIC exceeding 3 mg/g dry weight was indicative of hepatic iron overload [48]. The value of 20 ms was used as a “conservative” normal value for all 16 segments and the global T2* value [28,49].

For the standard assessment of biventricular function parameters, steady-state free precession (SSFP) cine images were obtained in consecutive short-axis slices encompassing the ventricles and three long-axis slices (two, three, and four chamber views) [50,51]. SSFP images were analyzed in a standard way using commercially available clinical workstations, either MASS software (Medis, Leiden, The Netherlands) or cmr42 (Circle Cardiovascular Imaging Inc., Calgary, Alberta, Canada) [52]. In brief, the endocardial and epicardial borders were manually delineated by an experienced observer on consecutive short-axis cine images at end-diastole and end-systole. End-diastolic volume and end-systolic volume were calculated using Simpson’s rule, without any geometric assumptions. Ejection fraction was determined by dividing the stroke volume (difference between end-diastolic volume and end-systolic volume) by the end-diastolic volume. The left ventricular mass was derived by multiplying muscle volume by its density (1.05 g/cm^3^). The papillary muscles were delineated and included in the myocardial mass, and the interventricular septum was considered part of the LV. Left and right atrial areas were quantified from the four-chamber view at ventricular end-systole. Biventricular volumes, LV mass, and bi-atrial areas were indexed to the body surface area (BSA), derived using the variation of the Dubois and Dubois formula [53].

For the detection of focal or replacement myocardial fibrosis, late gadolinium enhancement (LGE) images in both short- and long-axis views were acquired 8–18 min following intravenous administration of standard dose (0.2 mmol/kg) of Gadobutrol (Gadovist^®^; Bayer; Berlin, Germany) using a fast gradient-echo inversion recovery sequence [54]. In case of patient refusal or the presence of renal failure (glomerular filtration rate < 30 mL/min/1.73 m^2^), LGE images were not acquired. LGE images were visually evaluated to identify the presence and location of LGE. To exclude artifacts, LGE was considered present only if detected in two orthogonal views.

### 2.3. Biochemical Analysis

Biochemical tests were conducted at the local laboratories of the participating thalassemia centers using standardized, commercially available assay kits. For each participant, the average hemoglobin and serum ferritin levels from the 12 months prior to the CMR scan were determined and included in the analysis.

### 2.4. Statistical Analysis

Data analysis was performed by using SPSS version 27.0 (IBM Corp, Armonk, NY, USA) statistical package.

The normality of the distribution of the parameters was evaluated using the Kolmogorov-Smirnov test or the Shapiro-Wilk test for a sample size ≤ 50.

Means and standard deviations (SD) were used to describe continuous variables, whereas frequencies and percentages were reported for categorical variables.

The inter-group comparison for the continuous variables at baseline and the changes from baseline (differences between values at follow-up and baseline MRI scans) was carried out using the independent-samples t-test in the case of normal distribution or the Wilcoxon rank-sum test for non-normally distributed data. The comparison between categorical variables was made with the chi-square or Fisher’s exact test, as appropriate.

Analysis of covariance (ANCOVA) was employed to adjust for variables that differed significantly between the two groups at baseline and were significantly related to the dependent variable. A covariate was included if the variable showed both a significant difference between groups and a significant association with the outcome under evaluation. When necessary, outcome variables were log-transformed to achieve normality of residuals and homogeneity of variance.

The association between continuous variables was assessed using Pearson’s test or Spearman’s test, as appropriate based on data distribution.

Depending on the distribution of the variables of interest (normal or not), a paired Student t-test or Wilcoxon signed-rank test was performed to detect significant differences between baseline and follow-up values.

Two-tailed *p*-values were used for all analyses, and a threshold of *p* < 0.05 was considered statistically significant.

## 3. Results

### 3.1. Comparison of Baseline Data

The clinically and instrumentally relevant baseline findings in the two groups (NTDT vs. neo-TDT) are summarized in Table 1.

Age and sex were comparable between the two groups, while neo-TDT patients were more frequently splenectomized (*p* = 0.008) and more often receiving chelation therapy (*p* < 0.0001). Among patients who had undergone splenectomy, those in the neo-TDT group had a significantly younger age at the time of splenectomy compared to those in the NTDT group (17.05 ± 11.79 years vs. 24.20 ± 9.47 years, *p* = 0.026).

Neo-TDT patients showed significantly higher serum hemoglobin levels (*p* = 0.003), but no difference between the two groups was detected in serum ferritin levels.

Significantly lower hepatic iron levels were present in the neo-TDT group (*p* = 0.002), while cardiac iron levels were comparable between the two groups.

Biventricular volumes and LV mass index tended to be lower in neo-TDT patients compared to NTDT patients, but the difference was significant only for the LV end-diastolic volume index (*p* = 0.034). The neo-TDT group had a significantly lower cardiac index (*p* = 0.040), while no difference was observed in biventricular ejection fractions. Bi-atrial areas were comparable between the two groups.

The contrast medium was administered in 50.0% of NDT patients and 32.8% of neo-TDT patients (*p* = 0.108). The frequency of replacement myocardial fibrosis was comparable between the two groups. The septum was involved in all cases, and all patients had a non-ischemic LGE pattern (subendocardial or junctional).

### 3.2. MRI Changes for NTDT Patients

At the follow-up, no significant changes in mean hemoglobin levels (mean difference: −0.11 ± 0.53 g/dL, *p* = 0.408) and in mean serum ferritin levels (mean difference: −74.17 ± 395.83 ng/mL, *p* = 0.400) were found.

The difference in MRI parameters between the two MRI scans for NTDT patients is shown in Table 2.

No significant changes in MRI LIC values and global heart T2* values were detected. Out of the 24 patients with hepatic iron overload at the baseline, only 4 (16.7%) showed a normal MRI LIC at the follow-up MRI. The 50% of patients with a baseline MRI LIC < 3 mg/g dw became pathological at the follow-up MRI. The patient with significant myocardial iron overload (global heart T2* < 20 ms) at baseline showed a normal global heart T2* at the follow-up. At the baseline MRI, this patient was not chelated, but she started the chelation therapy (deferasirox) after that. All the 31 patients without significant myocardial iron overload at the baseline remained free of significant cardiac iron also at the follow-up MRI.

Biventricular volumes, LV mass index, and cardiac index increased at the follow-up, but the changes were not significant. Biventricular ejection fractions and bi-atrial areas remained almost unchanged.

There was no correlation between baseline serum hemoglobin levels and changes in LV function parameters, RV function parameters, or bi-atrial areas (Table 3).

At the follow-up, there were no new occurrences of replacement myocardial fibrosis.

### 3.3. MRI Changes for Neo-TDT Patients

At the follow-up, no significant changes in mean hemoglobin levels (mean difference: 0.07 ± 0.25 g/dL, *p* = 0.078) and in mean serum ferritin levels (mean difference: −21.37 ± 371.39 ng/mL, *p* = 0.837) were found.

The difference in MRI parameters between the two MRI scans for neo-TDT patients is shown in Table 4.

No significant changes in MRI LIC values and global heart T2* values were detected. Out of the 21 patients with hepatic iron overload at the baseline, 7 (33.3%) showed a normal MRI LIC at the follow-up MRI. Among the 37 patients without baseline hepatic iron overload, 9 (24.3%) showed hepatic iron overload at the follow-up. At both the MRI scans, all patients showed a normal global heart T2* value.

At the follow-up, a significant decrease in LV stroke volume index and cardiac index (*p* = 0.036 and *p* = 0.031, respectively) and RV end-diastolic volume index and stroke volume index was detected (*p* = 0.034 and *p* = 0.033, respectively). Biventricular ejection fractions, LV mass index, and bi-atrial areas were comparable between the two scans.

There was no correlation between baseline serum hemoglobin levels and changes in LV function parameters, RV function parameters, or bi-atrial areas (Table 3).

At the follow-up, there were no new occurrences of replacement myocardial fibrosis.

### 3.4. Inter-Group Comparison

The comparison of mean changes from baseline in MRI parameters between NTDT and neo-TDT patients is shown in Table 5.

The changes in MRI LIC and global heart T2* values were not significantly different between the two groups.

After adjustment for the baseline LV end-diastolic volume index, there was a statistically significant difference in the change in LV end-diastolic volume index between the two groups (*p* = 0.011). The changes in biventricular stroke volume indexes and the RV end-diastolic volume index were significantly different between the two groups (LV stroke volume index: *p* = 0.006, RV stroke volume index: *p* = 0.018, and RV end-diastolic volume index: *p* = 0.005), with all these parameters increased in the NTDT group and decreased in the neo-TDT group. The difference in the mean change in cardiac index (positive value for the NTDT group and negative value for the neo-TDT group, *p* = 0.009) remained significant also after the correction for the baseline value (*p* < 0.0001). The changes in biventricular end-systolic volume indexes, LV mass index, biventricular ejection fractions, and bi-atrial areas were comparable between the two groups.

## 4. Discussion

We longitudinally studied hepatic and cardiac siderosis and cardiac size and function in NTDT and neo-TDT patients.

In both patient groups, mean hepatic and cardiac iron levels were similar between the two MRI scans. Nevertheless, the comparison of the frequency of hepatic iron overload revealed important findings. Compared to the neo-TDT group, the NTDT group had a significantly higher prevalence of hepatic IO at the baseline and was characterized by a lower proportion of patients with hepatic IO at the baseline and a normal LIC at the follow-up MRI (16.7% vs. 33.3%) and a higher proportion of patients who developed hepatic IO at the follow-up (50.0% vs. 24.3%). The increased hepatic iron burden in NTDT patients can be attributed to the reduced frequency of iron chelation therapy.

The apparent discrepancy with previous studies, which reported increased cardiac iron levels in the neo-TDT group [33,37] may be attributed to the small sample size of this study. Furthermore, both patient groups maintained low cardiac iron levels, making it difficult to detect significant differences between them. Notably, in the single patient who presented with baseline cardiac iron overload, the initiation of chelation therapy was effective in reducing the cardiac T2* to a normal level, confirming the potential for treatment to manage and reverse cardiac iron overload [55,56,57,58,59].

At baseline, patients with NTDT showed more pronounced ventricular dilation compared to those with neo-TDT, suggesting, in line with previous studies [33,37], that the absence of regular transfusions may exacerbate the ventricular enlargement. The small sample size limited the statistical significance of most clinical parameters related to LV and RV remodeling. The follow-up MRI scans revealed a trend for worsening (increase) in biventricular dimensions for the NTDT group, which could be indicative of progressive cardiac dilation over time due to chronic anemia. In contrast, the neo-TDT group exhibited improvements in all cardiac size parameters, which reflected enhanced cardiac efficiency and better hemodynamic compensation. The decrease was significant for the biventricular stroke volume indexes, the cardiac index, and the RV end-diastolic volume index. The inter-group comparison showed significant differences in the changes of biventricular end-diastolic volume indexes and stroke volume indexes, and the cardiac index. Overall, our findings suggest that regular blood transfusions effectively reduce the negative hemodynamic effects of anemia, improving both short-term cardiac remodeling and providing lasting benefits in the heart’s structural adaptation over time.

Interestingly, we observed no significant differences in baseline values or the changes in biventricular ejection fractions between the two groups. The decline in myocardial systolic function is likely a gradual process, potentially requiring prolonged periods of high cardiac output to manifest. On the other side, a recent study conducted in patients with iron deficiency anemia showed that LV dysfunction can occur early in the progression of myocardial damage, even when the LV ejection fraction remains within normal limits [60]. Indeed, while the LV ejection fraction is the most commonly used index of LV systolic function in clinical practice, it has known limitations [61,62] and it often remains preserved until myocardial dysfunction reaches a more advanced stage, failing to detect early or subclinical dysfunction [63,64,65,66].

In our study, the mean serum hemoglobin at baseline was not correlated with baseline values or changes in biventricular volumes, likely because it does not fully reflect the long-term severity of anemia. Additionally, in the neo-TDT group, the well-controlled pre-transfusion hemoglobin levels make it challenging to identify a correlation, due to high intra-subject variability and the fact that MRI scans were taken at different points during the transfusion cycle. Although cardiac function generally remains stable before and after transfusions, ventricular volumes tend to decrease shortly after a blood transfusion [67]. However, the correction of anemia status in neo-TDT patients is not clearly indicated by the pre-transfusion hemoglobin level alone, as it depends on the post-transfusion hemoglobin level achieved and the transfusion interval. Moreover, the mean hemoglobin level reached is difficult to extrapolate.

### Limitations

This study has several limitations that should be acknowledged.

The main limitation of our study is the small sample size, which limits the statistical power and hampers drawing definitive conclusions or making broader inferences about the observed trends.

Another important factor to consider is the individual variability in transfusion regimens and iron chelation therapy, which may affect cardiac outcomes differently in neo-TDT patients. Nnon-“real-life” studies with standardized treatment protocols could help refine our understanding of these effects. Larger studies would allow for more robust statistical analyses, enhance the reliability of the results, and provide a clearer understanding. Additionally, long-term follow-up studies are needed to determine whether the effects of transfusion therapy on cardiac size and function in neo-TDT patients persist over time, as well as to further evaluate potential late-onset complications in patients with NTDT.

Myocardial strain is recognized as a more sensitive marker of myocardial dysfunction than ejection fraction [68]. Strain quantification can be performed using dedicated CMR feature-tracking software applied to routine cine images; however, these tools are not uniformly available across all MRI centers within the E-MIOT Network.

## 5. Conclusions

Our study highlights the distinct patterns of cardiac remodeling observed in NTDT and neo-TDT patients. NTDT patients show more pronounced increases in ventricular volumes and cardiac index, reflecting the natural progression of the disease in the absence of the beneficial effects of transfusion therapy on cardiac remodeling. Our findings underscore the importance of careful monitoring using MRI and the potential need for interventions in NTDT patients to mitigate the long-term cardiac consequences of anemia. Additionally, this study provides a basis for exploring the impact of emerging hemoglobin-boosting treatments, such as luspatercept and mitapivat, on cardiac size and function parameters in NTDT patients.

## Figures and Tables

**Table 1:** Comparison of baseline demographic, clinical, and MRI data.

	NTDT Patients (N = 32)	Neo-TDT Patients (N = 58)	*p*-Value
Age (years)	42.78 ± 12.62	44.08 ± 14.13	0.666
Female gender, N (%)	17 (53.1)	27 (46.6)	0.550
Splenectomy, N (%)	19 (59.4)	49 (84.5)	0.008
On chelation therapy, N (%)	13 (40.6)	57 (98.3)	<0.0001
Serum hemoglobin (g/dL)	8.99 ± 0.92	9.62 ± 0.62	0.003
Mean serum ferritin (ng/mL)	575.21 ± 631.42	753.95 ± 688.94	0.077
MRI LIC (mg/g dL)	7.23 ± 7.42	4.86 ± 7.56	0.002
Hepatic iron overload, N (%)	24 (75.0)	21 (36.2)	<0.0001
Global heart T2* (ms)	40.27 ± 6.45	39.13 ± 4.58	0.246
Global heart T2* < 20 ms, N (%)	1 (3.1)	0 (0.0)	0.356
Al least one cardiac segment with T2* < 20 ms, N (%)	3 (9.4)	4 (6.9)	0.696
LV end-diastolic volume index (mL/m^2^)	94.19 ± 17.14	86.34 ± 16.22	0.034
LV end-systolic volume index (mL/m^2^)	35.28 ± 11.08	31.30 ± 11.72	0.078
LV stroke volume index (mL/m^2^)	58.63 ± 9.49	55.46 ± 10.08	0.148
LV mass index (g/m^2^)	62.03 ± 12.61	58.02 ± 11.72	0.134
LV cardiac index (L/min/m^2^)	4.08 ± 0.87	3.67 ± 0.89	0.040
LV ejection fraction (%)	63.00 ± 5.74	65.29 ± 7.31	0.129
RV end-diastolic volume index (mL/m^2^)	88.92 ± 19.19	82.53 ± 16.66	0.103
RV end-systolic volume index (mL/m^2^)	35.29 ± 14.69	29.16 ± 9.58	0.059
RV stroke volume index (mL/m^2^)	55.48 ±11.22	53.13 ± 10.74	0.331
RV ejection fraction (%)	62.50 ± 5.85	64.21 ± 6.46	0.218
Left atrial area index (cm^2^/m^2^)	13.74 ± 2.59	13.12 ± 2.48	0.526
Right atrial area index (cm^2^/m^2^)	12.54 ± 2.44	12.08 ± 1.95	0.420
Replacement myocardial fibrosis, N (%)	4/16 (25.0)	4/19 (21.1)	0.782

LIC = liver iron concentration, LV = left ventricular, MRI = magnetic resonance imaging, N = number, NTDT = non-transfusion dependent thalassemia, RV = right ventricular, TDT = transfusion dependent thalassemia.

**Table 2:** Changes of MRI parameters between baseline and follow-up MRI scans in NTDT patients.

	Baseline MRI	Follow-Up MRI	*p*-Value
MRI LIC (mg/g dw)	7.23 ± 7.42	6.25 ± 4.63	0.128
Global heart T2* (ms)	40.27 ± 6.45	41.84 ± 5.16	0.331
LV end-diastolic volume index (mL/m^2^)	94.19 ± 17.14	96.69 ± 16.61	0.204
LV end-systolic volume index (mL/m^2^)	35.28 ± 11.08	35.81 ± 10.39	0.722
LV stroke volume index (mL/m^2^)	58.63 ± 9.49	61.35 ± 9.85	0.139
LV mass index (g/m^2^)	62.03 ± 12.61	64.04 ± 10.94	0.469
LV cardiac index (L/min/m^2^)	4.08 ± 0.87	4.32 ± 0.87	0.107
LV ejection fraction (%)	63.00 ± 5.74	64.00 ± 4.98	0.408
RV end-diastolic volume index (mL/m^2^)	88.92 ± 19.19	93.75 ± 19.29	0.063
RV end-systolic volume index (mL/m^2^)	35.29 ± 14.69	35.97 ± 10.83	0.155
RV stroke volume index (mL/m^2^)	55.48 ± 11.22	58.18 ± 10.35	0.187
RV ejection fraction (%)	62.50 ± 5.85	62.34 ± 5.21	0.874
Left atrial area index (cm^2^/m^2^)	13.74 ± 2.59	13.97 ± 3.68	0.738
Right atrial area index (cm^2^/m^2^)	12.54 ± 2.44	12.78 ± 2.97	0.454

LIC = liver iron concentration, LV = left ventricular, MRI = magnetic resonance imaging, RV = right ventricular.

**Table 3:** Association of baseline serum hemoglobin levels with changes between baseline and follow-up MRI scans in biventricular function parameters and bi-atrial areas.

	NTDT Patients (N = 32)	Neo-TDT Patients (N = 58)
	Correlation (r, *p*-Value) with Baseline Serum Hemoglobin Levels	
Diff LV end-diastolic volume index	r = −0.003, *p* = 0.989	r = −0.169, *p* = 0.231
Diff LV end-systolic volume index	r = −0.282, *p* = 0.154	r = 0.030, *p* = 0.834
Diff LV stroke volume index	r = 0.350, *p* = 0.073	r = −0.280, *p* = 0.084
Diff LV mass index	r = −0.330, *p* = 0.092	r = 0.004, *p* = 0.978
Diff LV cardiac index	r = 0.225, *p* = 0.260	r = −0.086, *p* = 0.545
Diff LV ejection fraction	r = 0.179, *p* = 0.074	r = −0.045, *p* = 0.574
Diff RV end-diastolic volume index	r = −0.271, *p* = 0.171	r = 0.062, *p* = 0.661
Diff RV end-systolic volume index	r = 0.140, *p* = 0.485	r = −0.077, *p* = 0.586
Diff RV stroke volume index	r = 0.163, *p* = 0416	r = −0.216, *p* = 0.125
Diff RV ejection fraction	r = 0.310, *p* = 0.116	r = −0.084, *p* = 0.556
Diff left atrial area index	r = −0.182, *p* = 0.430	r = −0.110, *p* = 0.523
Diff right atrial area index	r = 0.148, *p* = 0.522	r = −0.283, *p* = 0.094

Diff = difference, LV = left ventricular, N = number, NTDT = non-transfusion dependent thalassemia, RV = right ventricular, TDT = transfusion dependent thalassemia.

**Table 4:** Changes of MRI parameters between baseline and follow-up MRI scans in neo-TDT patients.

	Baseline MRI	Follow-Up MRI	*p*-Value
MRI LIC (mg/g dw)	4.86 ± 7.56	4.45 ± 6.53	0.792
Global heart T2* (ms)	39.13 ± 4.58	39.21 ± 5.53	0.941
LV end-diastolic volume index (mL/m^2^)	86.34 ± 16.22	84.24 ± 16.65	0.195
LV end-systolic volume index (mL/m^2^)	31.30 ± 11.72	30.83 ± 9.85	0.721
LV stroke volume index (mL/m^2^)	55.46 ± 10.08	54.21 ± 13.51	0.036
LV mass index (g/m^2^)	58.02 ± 11.72	58.78 ± 10.63	0.528
LV cardiac index (L/min/m^2^)	3.67 ± 0.89	3.45 ± 0.97	0.031
LV ejection fraction (%)	65.29 ± 7.31	63.95 ± 7.15	0.098
RV end-diastolic volume index (mL/m^2^)	82.53 ± 16.66	78.63 ± 18.49	0.034
RV end-systolic volume index (mL/m^2^)	29.16 ± 9.57	29.31 ± 8.65	0.929
RV stroke volume index (mL/m^2^)	53.13 ± 10.74	50.14 ± 11.49	0.033
RV ejection fraction (%)	64.21 ± 6.46	62.91 ± 6.17	0.099
Left atrial area index (cm^2^/m^2^)	13.12 ± 2.48	13.54 ± 2.49	0.188
Right atrial area index (cm^2^/m^2^)	12.08 ± 1.95	12.14 ± 2.02	0.844

LIC = liver iron concentration, LV = left ventricular, MRI = magnetic resonance imaging, RV = right ventricular.

**Table 5:** Comparison of mean changes from baseline in MRI parameters between NTDT and neo-TDT patients.

	NTDT Patients (N = 32)	Neo-TDT Patients (N = 58)	*p*-Value
Mean Diff MRI LIC (mg/g dw)	−0.97 ± 5.09	−0.41 ± 3.64	0.433
Mean Diff Global heart T2* (ms)	1.58 ± 5.70	0.08 ± 4.30	0.316
Mean Diff LV end-diastolic volume index (mL/m^2^)	2.50 ± 10.89	−2.10 ± 12.22	0.079
Mean Diff LV end-systolic volume index (mL/m^2^)	0.53 ± 8.33	−0.47 ± 10.01	0.856
Mean Diff LV stroke volume index (mL/m^2^)	2.72 ± 10.13	−1.25 ± 10.99	0.006
Mean Diff LV mass index (g/m^2^)	2.01 ± 12.45	0.76 ± 9.11	0.627
Mean Diff LV cardiac index (l/min/m^2^)	0.24 ± 0.83	−0.22 ± 0.76	0.009
Mean Diff LV ejection fraction (%)	1.00 ± 6.74	−1.34 ± 5.71	0.102
Mean Diff RV end-diastolic volume index (mL/m^2^)	4.83 ± 4.18	−3.91 ± 13.67	0.005
Mean Diff RV end-systolic volume index (mL/m^2^)	0.68 ± 9.91	0.14 ± 5.57	0.742
Mean Diff RV stroke volume index (mL/m^2^)	2.70 ± 11.32	−2.99 ± 10.43	0.018
Mean Diff RV ejection fraction (%)	−0.16 ± 5.51	−1.29 ± 5.87	0.403
Mean Diff left atrial area index (cm^2^/m^2^)	0.23 ± 3.22	0.42 ± 2.03	0.554
Mean Diff right atrial area index (cm^2^/m^2^)	0.25 ± 1.53	0.06 ± 2.09	0.484

Diff = difference, LIC = liver iron concentration, LV = left ventricular, MRI = magnetic resonance imaging, N = number, NTDT = non-transfusion dependent thalassemia, RV = right ventricular, TDT = transfusion dependent thalassemia.

## Data Availability

Data presented in this study are available on request from the corresponding author. The data are not publicly available due to privacy concerns.
